# Reducing salt intake with umami: A secondary analysis of data in the UK National Diet and Nutrition Survey

**DOI:** 10.1002/fsn3.3121

**Published:** 2022-11-12

**Authors:** Haruyo Nakamura, Takayuki Kawashima, Lisa Yamasaki, Kaung Suu Lwin, Akifumi Eguchi, Hitomi Hayabuchi, Yuta Tanoe, Shiori Tanaka, Daisuke Yoneoka, Cyrus Ghaznavi, Hisayuki Uneyama, Kenji Shibuya, Shuhei Nomura

**Affiliations:** ^1^ Tokyo Foundation for Policy Research Tokyo Japan; ^2^ Department of Mathematical and Computing Science Tokyo Institute of Technology Tokyo Japan; ^3^ Department of Global Health Policy, Graduate School of Medicine The University of Tokyo Tokyo Japan; ^4^ School of Medicine Nagasaki University Nagasaki Japan; ^5^ Centre for Preventive Medical Sciences Chiba University Chiba Japan; ^6^ Graduate School of Health and Environmental Sciences Fukuoka Women's University Fukuoka Japan; ^7^ Institute for Business and Finance Waseda University Tokyo Japan; ^8^ Division of Prevention National Cancer Center Institute for Cancer Control Tokyo Japan; ^9^ Infectious Disease Surveillance Center at the National Institute of Infectious Diseases Tokyo Japan; ^10^ Department of Health Policy and Management, School of Medicine Keio University Tokyo Japan; ^11^ Medical Education Program Washington University School of Medicine in St. Louis St. Louis Missouri USA; ^12^ Ajinomoto Co., Inc. Tokyo Japan

**Keywords:** salt, sodium, umami, United Kingdom

## Abstract

Reducing sodium content in foods is an important public health measure to reduce salt intake and decrease the incidence of noncommunicable diseases, such as cardiovascular disease and chronic kidney disease. This study quantified the amount of salt intake that could potentially be reduced by using umami substances, including glutamate, inosinate, and guanylate, without compromising taste, for adults in the United Kingdom (UK). We used data comprised of 1834 adults aged 20 years and over from the National Diet and Nutrition Survey (NDNS RP) 2016/2017–2018/2019. Four hypothetical scenarios in which the market share of low‐sodium foods accounts for 0%, 30%, 60%, or 90% of consumed products were considered in the analyses. Per capita daily salt intake corresponding to the NDNS RP food groups was calculated for each scenario, and the salt intake was aggregated by gender and age groups. Replacing salt with umami substances could help UK adults reduce daily salt intake by 9.09%–18.59% (9.21%–18.43% for women; 8.83%–19.43% for men), which is equivalent to 0.45–0.92 g/day of salt reduction (0.41–0.82 g/day for women; 0.50–1.10 g/day for men). The use of umami substances may serve as one method for the UK government to encourage salt intake reduction, particularly in the context of food product reformulation, as 80% of salt consumed in the country comes from processed foods. Empirical studies with sensory evaluation should be conducted to confirm consumer tolerance. The food industry should also be engaged in conversations regarding the addition of umami to food products in the United Kingdom.

## Graphical abstract

The use of umami substances may serve as one method for the UK government to encourage salt intake reduction. Replacing salt with umami substances could help UK adults reduce daily salt intake by 9.10‐18.59% (9.21‐18.43% for women; 8.83‐19.43% for men), which is equivalent to 0.45‐0.92g/day of salt reduction (0.41‐0.82g/day for women; 0.50‐1.10g/day for men).

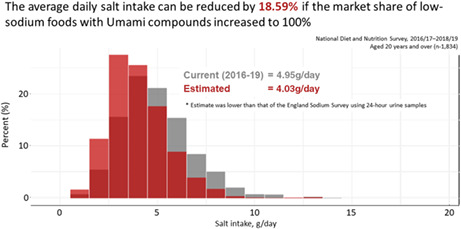

## 1 INTRODUCTION

In May 2022, 70 health and scientific organizations advocated for sodium (salt) reduction to be made a high global priority and for all nations to develop effective programs to reduce sodium intake to recommended levels (Campbell et al., 2022). High sodium consumption contributes to high blood pressure (Aburto et al., 2013; World Health Organization, 2020), which increases the risk of cardiovascular diseases (Bibbins‐Domingo et al., 2010; British Heart Foundation, 2022; Brown et al., 2009; Cogswell et al., 2016; Singh et al., 2013; Thomopoulos et al., 2014), chronic kidney disease (Xie et al., 2016), and gastric cancer (D'Elia et al., 2012; World Cancer Research Fund & American Institute for Cancer Research, 2007). In 2019, approximately 1.9 million deaths worldwide were attributed to a high salt diet (GBD 2019 Risk Factors Collaborators, 2020), and the number of deaths increased by 42.8% from 1990 to 2019 (Chen et al., 2021). An estimated 2.5 million deaths could be prevented each year if global salt consumption were reduced to recommended levels (World Health Organization, 2020). Salt intake reduction is also known to be one of the most cost‐effective or even cost‐saving noncommunicable disease (NCD) control measures (Cobiac et al., 2010). The reduction of salt intake by 30% between 2011 and 2025 is one of the nine targets in the NCD Global Monitoring Framework initiated by WHO in 2013 (World Health Organization, 2014); however, no country has yet to achieve this target to date (Development Initiatives, 2020).

As of 2017, approximately 31% of adults aged 16 years or older in England had high blood pressure, which is the third most common risk factor for premature death and disability in the country (Public Health England, 2020a). The United Kingdom (UK) government initiated a public health campaign to encourage salt intake reduction in the early 1990s. In 2003, the UK Scientific Advisory Committee on Nutrition (SACN) published its report recommending a maximum salt intake of 6 g/day or less for adults (Sutherland et al., 2013), which was comparable with recommendations from WHO (5 g/day) and those from the United States and Canada (5.85 g/day) (Public Health England, 2020a). Following the publication of SACN, the UK government began a program aiming to reduce salt intake across the population (Sutherland et al., 2013). The UK Food and Standards Agency (FSA) and the Department of Health proposed two strategies to achieve salt intake reduction: (1) a reduction in the salt content of processed foods through engagement with the food industry and (2) an increase in consumer awareness of the impact of salt on health via a public awareness campaign (He et al., 2014). The UK salt reduction strategy was initially successful (Brinsden et al., 2013; Charlton et al., 2014; Gressier et al., 2021; He et al., 2014; Pombo‐Rodrigues et al., 2017; Sutherland et al., 2013; Tan et al., 2019), and the mean population salt intake was reduced from 9.5 g/day in 2001 to 8.1 g/day in 2011 (He et al., 2014). However, recent public health policies have had a tendency to focus on sugar and calorie reduction rather than salt. Consequently, the latest trend analyses suggested that there have been no further significant advancement in salt reduction since 2008/2009 (Public Health England, 2020a). The mean population salt intake for adults aged 19–64 years was estimated to be 8.4 g/day in 2018/2019, 40% higher than the government recommendation (Public Health England, 2020a), and 68% higher than that of WHO (World Health Organization, 2012). Stronger action is needed to reduce the risk of cardiovascular disease and associated costs for health services and society.

In recent years, the replacement of sodium chloride with “umami” has been discussed as a healthy and natural solution to reduce salt intake while maintaining palatability (Hayabuchi et al., 2020; Nomura et al., 2021; Umeki et al., 2021; Yamaguchi & Takahashi, 1984). Umami, which is translated as "savory" in Japanese, is induced by monosodium glutamate (MSG) and 5′‐ribonucleotides, such as guanosine monophosphate and inosine monophosphate. Even though umami is considered the fifth basic taste sensation, following saltiness, sweetness, bitterness, and acidity (Beauchamp, 2009), few studies have been conducted to empirically evaluate the impact of umami on salt reduction at the population level. Recently, it was reported in Japan that the daily salt intake per adult could be reduced by up to 2.22 g if umami substances were incorporated into selected marketed foods (Tanaka et al., 2021).

In this study, we examined how the use of umami affects the daily salt intake of UK adults using data from the National Diet and Nutrition Survey Rolling Programme (NDNS RP) that is carried out by a consortium comprising NatCen Social Research and the National Institute of Health Research Cambridge Biomedical Research Center (Public Health England, 2020b). The results of this study will serve as a first step in considering alternative methods of reducing daily salt intake by addressing upstream food production processes.

## 2 METHODS

### 2.1 Study design and participants

We used anonymous, secondary, open data from the NDNS RP for the general population aged 1.5 years and over living in private households in the UK between April 2016 and June 2019 (Public Health England, 2020b). The NDNS RP is a cross‐sectional survey with a stratified, multistage probability sample design, which collects a four‐day food diary. Every participant writes down everything that he/she ate and drank for the designated 4 days. At the time of the first visit to a household, a laptop computer automatically selects four consecutive days as the diary recording period for each household such that all days of the week are evenly represented in the survey. Interviewers collected demographic information on the household members, including their gender and ages. On the second day of the diary recording, a check‐up visit is arranged. The diary is picked up within 3 days after the final day of diary recording. The questionnaires, datasets, and all related documents for the NDNS RP are available on the UK government website (Public Health England, 2021a).

### 2.2 Food and sodium intake data

All individual ingredients of a homemade recipe as reported in the food diary, or components of the purchased product as described on the food packaging, were coded with their respective food codes and linked together under the appropriate Recipe Food Group, which highlights that those food codes were consumed together in one composite dish. The consumed amount of all foods is described in grams.

The nutrient intakes (e.g., sodium) were calculated per 100 g based on the NDNS nutrient databank (NDB), which is populated with information from the UK Composition of Foods Integrated Dataset (Public Health England, 2021b), and is supplemented by the FSA Food Recipes Database (MRC Human Nutrition Research, 2017) and manufacturers' data gathered through food labels and web information. The NDB is updated annually. Each food code in the NDB has a value assigned for 56 nutrients that is further disaggregated into 28 specific food components to ensure accurate reporting of specific food types in the NDNS RP. Each food code is assigned to a food subgroup, expressed as an integer with an alphabetical suffix, which is a food group level of greater detail than the main food groups (Public Health England, 2020b).

In this study, the average intake of each food group and the corresponding sodium intake from the 4‐day food diary was calculated and analyzed as a daily value. Salt equivalent intake (g) was defined as sodium (mg) × 2.54/1000. It is important to note that the sampling weight was not applied. This is because we aimed to evaluate the distribution of daily salt intake on an individual basis and to examine how much it would change before and after the incorporation of umami substances (Public Health England, 2021c).

### 2.3 Sodium reduction rate in various food products with the incorporation of umami substances

We searched PubMed from inception to April 6, 2022 for English‐language articles that estimate the potential sodium reduction rate by using umami substances with the search terms (“sodium intake” OR “salt intake” OR “sodium reduction” OR “salt reduction”) AND (“umami” OR “MSG” OR “monosodium glutamate” OR “inosinate” OR “CDG” OR “calcium diglutamate” OR “guanilate” OR “guanylate”). Based on previous studies and input from several food and nutrition experts (coauthors), we estimated salt reduction rates achieved by using umami substances for the NDNS RP food subgroups as listed in Table 1.

**TABLE 1:** Prior research examining sodium reduction due to incorporation of umami substances by NDB food code

Main group	Food subgroup	NDNS RP main food code	Sodium reduction rate (%)	References			Umami substance
Milk	1. Cheese	14	54–100	Rodrigues et al. (2014)	da Silva et al. (2014)		MSG
Meat	2. Sausage	22, 30	17–75	Woodward et al. (2003)	Ichikawa Chemical Institution (1984)	dos Santos et al. (2014)	MSG, CDG, Inosinate
	3. Chicken broth	***	11–38	Chi and Chen (1992)	Carter et al. (2011)	Wang, Tonnis, et al. (2019)	MSG, CDG
Fish	4. Salted fish	***	30–40	Ichikawa Chemical Institution (1984)			MSG, Inosinate
Legume	5. Miso	***	15–35	Ishida et al. (2011)	Yamasa Corporation (2014)		MSG, Inosinate, Guanilate
	6. Soy sauce	***	40–61	Kao Corporation (2006)	Ishida et al. (2011)	Kameda seika Co. Ltd (1997)	MSG, Inosinate, Guanilate
Grain	7. Snack	42	51	Buechler and Lee (2020)			MSG, Inosinate, Guanilate
Vegetable	8. Vegetable soup	***	17–40	Kremer et al. (2009)	Ball et al. (2002)		Glutamates, CDG
	9. Potato chips	38	30	Kongstad and Giacalone (2020)			MSG
	10. Salted vegetable	***	55	Tampei Pharmaceutical Co., Ltd (1985)			MSG
Oil	11. Butter	17	100	de Souza et al. (2013)			MSG

*Note*: *** refers to all food codes for which the first one or two digits correspond.

Abbreviations: CDG, calcium diglutamate; MSG, monosodium glutamate; NDB, The UK Nutrient Databank.

### 2.4 Estimating salt intake reduction with the incorporation of umami substances

As people in the UK already consume a certain amount of low‐sodium foods in their daily diets, we set four hypothetical scenarios in which the market share of low‐sodium products accounts for 0% (i.e., no low‐sodium foods are in the market), 30%, 60%, or 90% of food products.

For each of the major food groups, we calculated the amount of salt that could possibly be reduced for each of the four scenarios by gender and age group (20–29, 30–39, 40–49, 50–59, 60–69, 70–79, and 80+). The salt reduction rate for each NDNS RP food subgroup was expressed as an upper‐lower interval in Table 1, representing the range of possible salt reduction rates estimated from the literature. The upper and lower limits were then used to calculate the maximum and minimum possible salt reduction for each food subgroup at the individual level.

The following equations give the upper and lower limits of the *j*‐th food subgroup specific reduction in salt intake by using umami substances in the *i*‐th individual:

$$\begin{array}{c} \text{Upper reduction in salt intake of the}j\mathrm{th}\text{item under the}k\mathrm{th}\text{scenario in the}i\mathrm{th}\text{individual}\\ =S_{\mathrm{ij}}-S_{\mathrm{ij}}\times U_{j}\times \left(1-M_{k}\right), \end{array}$$

$$\begin{array}{c} \text{Lower reduction in salt intake of the}j\mathrm{th}\text{item under the}k\mathrm{th}\text{scenario in the}i\mathrm{th}\text{individual}\\ =S_{\mathrm{ij}}-S_{\mathrm{ij}}\times L_{j}\times \left(1-M_{k}\right), \end{array}$$

where *S*_*ij*_ refers to the current salt intake of the *j*‐th food subgroup in the *i*‐th participant; *U*_*j*_ and *L*_*j*_ refer to the upper and lower limits of the sodium reduction rate of the *j*‐th food subgroup, and *M*_*k*_ refers to the *k*‐th scenario of the market share of low‐sodium consumed products (denoted as *M*_*k*_ = 0, 0.3, 0.6 or 0.9 [*k* = 1, 2, 3 or 4, respectively]).

After calculating the individual‐level salt reduction for each scenario and food subgroup using the formula above, we aggregated the amount of salt that could be reduced per major group and calculated the average value per population. R version 4.0.5 was used for all analyses.

## 3 RESULTS

The NDNS RP 2016/2017–2018/2019 cohort comprises a total of 3558 individuals (1922 women and 1636 men) aged 1.5 years and over (mean age 30.59; standard deviation [SD] 24.49) who completed the diet diary for 4 (97%) or 3 days (3%). This analysis included those who were aged 20 years and over, a total of 1834 individuals (1076 women and 758 men), with a mean age of 50.79 (SD 17.22).

Table 2 shows the gender‐ and age group‐specific mean daily salt intake. Women had a lower salt intake than men across all age groups. The mean daily salt intake was highest among women aged 30–39 years (4.86 g/day) and men aged 20–29 years (6.32 g/day), but lowest among those aged 70–79 years for both women (3.98 g/day) and men (4.89 g/day). Salt intake tended to be higher among younger than older persons.

**TABLE 2:** Demographic characteristics of study participants and their current salt intake

Age (years)	Number of participants	Current mean salt intake (SD) (g/day)
Total population		
20–29	230	5.37 (2.31)
30–39	332	5.39 (2.13)
40–49	340	5.13 (1.85)
50–59	340	4.80 (1.78)
60–69	275	4.65 (1.66)
70–79	208	4.36 (1.46)
80+	109	4.49 (1.66)
All	1834	4.95 (1.91)
Women		
20–29	145	4.81 (1.91)
30–39	194	4.86 (1.84)
40–49	210	4.63 (1.51)
50–59	193	4.18 (1.37)
60–69	148	4.19 (1.34)
70–79	121	3.98 (1.28)
80+	65	4.13 (1.50)
All	1076	4.45 (1.60)
Men		
20–29	85	6.32 (2.63)
30–39	138	6.14 (2.27)
40–49	130	5.94 (2.05)
50–59	147	5.62 (1.92)
60–69	127	5.20 (1.82)
70–79	87	4.89 (1.52)
80+	44	5.01 (1.76)
All	758	5.66 (2.09)

Abbreviation: SD, standard deviation.

The estimated amount of total salt intake reduction for UK adults aged 20 years and over across four scenarios of low‐sodium product market shares – 0%, 30%, 60%, and 90% – was 0.45–0.92 g/day, 0.31–0.64 g/day, 0.18–0.37 g/day, and 0.045–0.092 g/day, respectively (Table 3).

**TABLE 3:** Estimated salt reduction by scenario and gender (g/day)

	Scenario 1 (current market share of low‐sodium products = 0%)	Scenario 2 (current market share of low‐sodium products = 30%)	Scenario 3 (current market share of low‐sodium products = 60%)	Scenario 4 (current market share of low‐sodium products = 90%)
Total population				
20–29	0.48–0.98	0.33–0.69	0.19–0.39	0.048–0.098
30–39	0.47–0.95	0.33–0.67	0.19–0.38	0.047–0.095
40–49	0.49–1.01	0.34–0.71	0.20–0.40	0.049–0.100
50–59	0.42–0.87	0.29–0.61	0.17–0.35	0.042–0.087
60–69	0.42–0.90	0.29–0.63	0.17–0.36	0.042–0.090
70–79	0.39–0.80	0.27–0.56	0.16–0.32	0.039–0.080
80+	0.42–0.85	0.30–0.60	0.17–0.34	0.042–0.085
All	0.45–0.92	0.31–0.64	0.18–0.37	0.045–0.092
Women				
20–29	0.43–0.86	0.30–0.60	0.17–0.34	0.043–0.086
30–39	0.45–0.88	0.31–0.62	0.18–0.35	0.045–0.088
40–49	0.44–0.89	0.31–0.62	0.18–0.36	0.044–0.089
50–59	0.37–0.75	0.26–0.52	0.15–0.30	0.037–0.075
60–69	0.40–0.82	0.28–0.58	0.16–0.33	0.040–0.082
70–79	0.37–0.70	0.26–0.49	0.15–0.28	0.037–0.070
80+	0.37–0.76	0.26–0.53	0.15–0.31	0.037–0.076
All	0.41–0.82	0.29–0.57	0.16–0.33	0.041–0.082
Men				
20–29	0.56–1.20	0.40–0.84	0.23–0.48	0.056–0.120
30–39	0.50–1.04	0.35–0.73	0.20–0.42	0.050–0.100
40–49	0.56–1.20	0.39–0.84	0.22–0.48	0.056–0.120
50–59	0.49–1.04	0.34–0.73	0.20–0.42	0.049–0.100
60–69	0.44–0.98	0.31–0.69	0.18–0.39	0.044–0.098
70–79	0.43–0.93	0.30–0.65	0.17–0.37	0.043–0.093
80+	0.50–0.98	0.35–0.69	0.20–0.39	0.050–0.098
All	0.50–1.10	0.35–0.74	0.20–0.42	0.050–0.110

Abbreviation: SD, standard deviation.

Table 4 shows lower‐upper mean salt intake reduction rates that could be achieved by incorporating umami substances in food products by gender and age group across the four scenarios. We found that 9.09%–18.59% of salt intake could be reduced by incorporating umami substances into UK adults' diets under Scenario 1. The reduction rates were 6.26%–12.93%, 3.64%–7.47%, 0.91%–1.86% for Scenarios 2, 3 and 4, respectively.

**TABLE 4:** Estimated salt reduction rate by scenario, gender, and age (%)

	Scenario 1 (current market share of low‐sodium products = 0%)	Scenario 2 (current market share of low‐sodium products = 30%)	Scenario 3 (current market share of low‐sodium products = 60%)	Scenario 4 (current market share of low‐sodium products = 90%)
Total population				
20–29	8.94–18.25	6.15–12.85	3.54–7.26	0.89–1.82
30–39	8.72–17.63	6.12–12.43	3.53–7.05	0.87–1.76
40–49	9.55–19.69	6.63–13.84	3.90–7.80	0.96–1.95
50–59	8.75–18.13	6.04–12.71	3.54–7.29	0.88–1.81
60–69	9.03–19.35	6.24–13.55	3.66–7.74	0.90–1.94
70–79	8.94–18.35	6.19–12.84	3.67–7.34	0.89–1.83
80+	9.35–18.93	6.68–13.36	3.79–7.57	0.94–1.89
All	9.09–18.59	6.26–12.93	3.64–7.47	0.91–1.86
Women				
20–29	8.94–17.88	6.24–12.47	3.53–7.07	0.89–1.79
30–39	9.26–18.11	6.38–12.76	3.70–7.20	0.93–1.81
40–49	9.50–19.22	6.70–13.39	3.89–7.78	0.95–1.92
50–59	8.85–17.94	6.22–12.44	3.59–7.18	0.89–1.79
60–69	9.55–19.57	6.68–13.84	3.82–7.88	0.95–1.96
70–79	9.30–17.59	6.53–12.31	3.77–7.04	0.93–1.76
80+	8.96–18.40	6.30–12.83	3.63–7.51	0.90–1.84
All	9.21–18.43	6.52–12.81	3.60–7.42	0.92–1.84
Men				
20–29	8.86–18.99	6.33–13.29	3.64–7.59	0.89–1.90
30–39	8.14–16.94	5.70–11.89	3.26–6.84	0.81–1.63
40–49	9.43–20.20	6.57–14.14	3.70–8.08	0.94–2.02
50–59	8.72–18.51	6.05–12.99	3.56–7.47	0.87–1.78
60–69	8.46–18.85	5.96–13.27	3.46–7.50	0.85–1.88
70–79	8.79–19.02	6.13–13.29	3.48–7.57	0.88–1.90
80+	9.98–19.56	6.99–13.77	3.99–7.78	1.00–1.96
All	8.83–19.43	6.18–13.07	3.53–7.42	0.88–1.94

## 4 DISCUSSION

In this study, we found that it is possible to reduce population‐level salt intake among UK adults aged 20 years and over by up to 9.09%–18.59% (9.21%–18.43% for women; 8.83%–19.43% for men), which is equivalent to 0.45–0.92 g/day of salt reduction (0.41–0.82 g/day for women; 0.50–1.10 g/day for men) without compromising palatability by incorporating umami substances into food products.

Our findings regarding salt reduction were comparable or slightly higher than that of a previous study based on US population data that suggested a potential range of salt intake reduction of 5.51%–10.54% (Wallace et al., 2019). The difference in the values of possible salt intake reduction is likely to related to differences in the types of foods consumed in the two countries.

Salt intake reduction has been strongly recommended by WHO to reduce blood pressure and the risk of NCDs (World Health Organization, 2012). While the UK government was among the first to launch a salt reduction campaign which was quite successful until the late 2000s (Charlton et al., 2014; He et al., 2014; Tan et al., 2019), more recent policies rarely focus on salt reduction at the population level (Burt et al., 2022; Huang et al., 2021). Consequently, no significant progress has been made since 2008/2009 (Public Health England, 2020a). Recently, a British NGO expressed concern about the lack of progress and sent a letter to the Prime Minister urging him to prioritize salt reduction as a highly impactful and cost‐effective public health intervention (Action on Salt, 2022). Our study suggests that the use of umami substances could be an alternative for the UK government to revive salt intake reduction efforts, as it has been demonstrated that low‐sodium food would be more palatable when a small amount of umami substances are added (Committee on Strategies to Reduce Sodium Intake Food and Nutrition Board, 2010; Hoppu et al., 2017; Precott, 2004).

The UK government may want to consider using umami substances in the course of food product reformulation. Although the proportion of adults who generally added salt at the table reportedly decreased from 40.1% in 1997 to 31.7% in 2007 (Sutherland et al., 2013), a more recent study found that the proportion of participants declaring they added salt did not change over time, that is, 50% in year 2008/2009 and 54% in year 2016/2017 (Gressier et al., 2021). On the other hand, several systematic reviews on strategies to improve diets have suggested that food product reformulation would effectively reduce sodium intake (Hyseni et al., 2017; McLaren et al., 2016), and it is estimated that 80% of salt consumed in the UK indeed comes from processed foods (Charlton et al., 2014; Gressier et al., 2021). Thus, it is important for the government to work together with industry to accelerate salt reduction efforts, and the use of umami is one such solution.

Although umami was first discovered by a Japanese scientist (Lindemann et al., 2002), he found the taste in tomato, asparagus, cheese, and meat when he was in Germany (Ninomiya, 2015). There is a variety of free amino acids, umami, found in European soup stocks. Indeed, umami substances can be used to replace salt in various kinds of food and it has been increasingly accepted worldwide (Halim et al., 2020; Jinap et al., 2016; Jinap & Hajeb, 2010; Simões do Couto Rosa et al., 2021). An example of a Western dish that could benefit from salt reduction is chicken pot pie, which invokes a comforting feeling but comes with a hefty amount of sodium. By replacing some of the salt with MSG, the sodium can be reduced by up to 25% while the umami is enhanced (Hedrick, 2022). MSG was once believed to cause side effects, such as headache and nausea. However, studies have found no consistent clinical data to support these claims (International Glutamate Information Service, 2022a, 2022b; Maluly et al., 2017) and the safety of MSG has been approved by official agencies worldwide, including those in Europe (EFSA Panel on Food additives and Nutrient Sources added to Food, 2015).

The salt content of vegetarian and vegan products is found to be higher than regular food products. A study on meat‐free alternatives found that the salt content was generally higher than that of meat, and 28% were reportedly even higher than the maximum salt reduction targets (Action on Salt, 2018). It is estimated that 9% of adults in the UK (4.9 million) are vegetarian or vegan, and another 8.8 million plan to go meat free in 2022 (Finder UK, 2022). As umami technology was developed in the context of Japan's plant‐based food culture, umami might be a valuable tool for the sustainable reduction of salt in vegetarian and vegan cuisine.

Our study has several strengths. First, this is the first study to quantify the amount of salt that could be reduced by incorporating umami substances into the diet of UK adults. Another strength of our study is that we used the NDNS RP data, which is comprised of a large, nationally representative sample, which allowed us to estimate the mean salt intake at the population level. Our study has several limitations. This study estimated the mean salt intake of UK adults in 2016–2019 to be 4.95 g/day (4.45 g/day for women and 5.66 g/day for men); our estimate was lower than that of the 2018/2019 England Sodium Survey, which estimated an arithmetic mean salt intake of 8.4 g/day for adults aged 19–64 years (6.8 g/day for women and 9.2 g/day for men) (Public Health England, 2020a). This difference is primarily attributed to two factors. First, the 2018/2019 England Sodium Survey used 24‐h urine samples to estimate mean salt intake while we used food diary data to estimate how much salt can be reduced from each food item. There is a risk of underreporting or misreporting in the food survey. Furthermore, previous research found higher sodium intake measured using urine excretion as compared to that measured with food surveys (Gressier et al., 2021). Second, it should be noted that salt added during cooking or at the table was not considered in this study, as the NDNS RP participants were not asked to write in their diaries whether they used additional salt (Gressier et al., 2021). Although the majority of salt intake is from processed foods, salt added during cooking or at the table remains a significant source of sodium consumption, with an estimated 15%–20% of dietary salt obtained from discretionary sources (Scientific Advisory Committee on Nutrition, 2003). Precaution should be taken that the mean salt intake estimated in our analysis might be lower than the actual amount. In addition, this study relied on existing literature to derive salt reduction rates by using umami substances. However, the evidence may be insufficient for some food groups. Furthermore, we assumed that the market share of low‐sodium food is the same across all food groups due to limited data availability. Divergence of low‐sodium product intake, depending on the place where the meal is served or prepared (home or restaurant) was also not considered in this study due to scarcity of data that properly examined these factors. Finally, we were unable to confirm the acceptability of umami substances among UK consumers (Miyaki et al., 2016; Wang, Zhang, et al., 2019). Future studies with sensory evaluation should be conducted to empirically confirm consumer tolerance. The food industry should also be engaged in conversations regarding the addition of umami substances to food products in the UK. This experiment in the UK may help reduce the high‐sodium‐related burden of diseases around the globe.

## 5 CONCLUSIONS

The incorporation of umami substances into certain foods could potentially reduce population‐level daily salt intake in the UK by up to 9.09%–18.59%, which is equivalent to 0.45–0.92 g/day of salt reduction without compromising taste. Although the results of our study should be interpreted with caution as we used food surveys rather than urinary sodium excretion to calculate salt intake, our findings suggest an alternative for the UK government to encourage salt intake reduction during upstream processes such as food production. Additional empirical studies with sensory evaluation should be conducted to confirm consumer tolerance of umami products, and the UK food industry should be engaged in discussions regarding umami's application in food production. Ultimately, the experiment in the UK is expected to help reduce the high sodium‐related burden of diseases around the globe.

## FUNDING INFORMATION

This article was partially supported by a joint research grant from Ajinomoto Co., Inc. H.U. is employed by the commercial funder, Ajinomoto Co., Inc. The commercial funder provided support in the form of salaries for H.U., but did not have any additional role in the decision to publish or the preparation of this manuscript. This article was also partly funded by a research grant from the Ministry of Education, Culture, Sports, Science and Technology of Japan (21H03203).

## CONFLICT OF INTEREST

K.S. report a grant from the Ajinomoto Co., Inc. H.U. declares that he is employed by Ajinomoto Co., Inc. and has no other competing interests. All other authors declare no competing interests.

## Data Availability

The data are publicly available at https://beta.ukdataservice.ac.uk/datacatalogue/studies/study?id=6533 (accessed on June 29, 2022).
